# B, O and N Codoped Biomass-Derived Hierarchical Porous Carbon for High-Performance Electrochemical Energy Storage

**DOI:** 10.3390/nano12101720

**Published:** 2022-05-18

**Authors:** Shuying Kong, Xinzhu Xiang, Binbin Jin, Xiaogang Guo, Huijun Wang, Guoqing Zhang, Huisheng Huang, Kui Cheng

**Affiliations:** 1 Chongqing Key Laboratory of Inorganic Special Functional Materials, College of Chemistry and Chemical Engineering, Yangtze Normal University, Chongqing 404100, China; xiangxinzhu6666@163.com (X.X.); bbjin001@126.com (B.J.); guoxiaogang0528@126.com (X.G.); wanghj@yznu.edu.cn (H.W.); yzhanggq@163.com (G.Z.); h.s.huang@hotmail.com (H.H.); 2 College of Engineering, Northeast Agricultural University, Harbin 150030, China

**Keywords:** supercapacitors, lithium-ion batteries, energy storage, biomass, porous carbon

## Abstract

High specific surface area, reasonable pore structure and heteroatom doping are beneficial to enhance charge storage, which all depend on the selection of precursors, activators and reasonable preparation methods. Here, B, O and N codoped biomass-derived hierarchical porous carbon was synthesized by using KCl/ZnCl_2_ as a combined activator and porogen and H_3_BO_3_ as both boron source and porogen. Moreover, the cheap, environmentally friendly and heteroatom-rich laver was used as a precursor, and impregnation and freeze-drying methods were used to make the biological cells of laver have sufficient contact with the activator so that the layer was deeply activated. The as-prepared carbon materials exhibit high surface area (1514.3 m^2^ g^−1^), three-dimensional (3D) interconnected hierarchical porous structure and abundant heteroatom doping. The synergistic effects of these properties promote the obtained carbon materials with excellent specific capacitance (382.5 F g^−1^ at 1 A g^−1^). The symmetric supercapacitor exhibits a maximum energy density of 29.2 W h kg^−1^ at a power density of 250 W kg^−1^ in 1 M Na_2_SO_4_, and the maximum energy density can reach to 51.3 W h kg^−1^ at a power density of 250 W kg^−1^ in 1 M BMIMBF_4_/AN. Moreover, the as-prepared carbon materials as anode for lithium-ion batteries possess high reversible capacity of 1497 mA h g^−1^ at 1 A g^−1^ and outstanding cycling stability (no decay after 2000 cycles).

## 1. Introduction

Nowadays, the energy crisis, environmental pollution and global warming are all related to the heavy dependence on nonrenewable resources (fossil energy such as coal and oil) in today’s society. Therefore, the development and utilization of green and sustainable energy (wind, solar, tidal energy, etc.) has become very urgent [1]. However, compared with nonrenewable energy, renewable energy cannot be directly stored on a large scale, so the development of efficient and stable sustainable energy-conversion and storage technology has become a research focus in the past few decades. A lot of research has been carried out on the development of storage systems with high energy density and power density. At present, energy-storage and conversion devices mainly include secondary batteries, fuel cells and supercapacitors [2,3,4]. Electrode material is a vital part of energy-storage devices, which can determine electrochemical performance. Therefore, the development of high-performance, low-cost, environmentally friendly electrode materials is the focus of current research. 

Carbon-based materials, owing to their advantages such as high intrinsic conductivity, simple preparation method, outstanding rate performance and excellent chemical stability, are widely used in commercial energy-storage devices [5,6,7]. Among the carbonaceous materials used as electrode materials, biomass-derived porous carbons exhibit outstanding performance [8,9,10]. Biomass is not only naturally abundant and cost-effective, but also produces carbon materials with 3D porous morphology and high specific surface area. The unique 3D microstructure of hierarchical porous carbons comprises macropores, mesopores and micropores. Micropores provide more electrolyte-ion-adsorption sites; macropores and mesopores can reduce the diffusion distance and diffusion resistance, respectively, which is conducive to the diffusion of the electrolyte, thereby increasing the power density of the electrode material [11]. Therefore, choosing a reasonable preparation method and activator to obtain hierarchical porous carbon is the focus of research. Up to now, the activators commonly used in carbon materials are KOH, HNO_3_, ZnCl_2_, FeCl_3_ and K_2_CO_3_ [12,13,14]. Among them, salt activators are less corrosive to instruments than strong acids and bases, which are widely used to prepare carbon materials with unique pore structure and high specific surface area. Moreover, the activated carbon prepared by the mixed activator owns more mesopores, macropores and specific surface area than the single activator, so it has higher electrochemical performance [15]. CoCl_2_/ZnCl_2_ mixed activator is applied to synthesize porous carbonaceous materials, which exhibits high surface area (1745–2257 m^2^g^−1^) and high specific capacitance (343 F g^−1^ at 0.5 A g^−1^) [16]. Wan et al. prepared a N and S codoped hierarchically porous carbon (RPHPC) using mix salts of ZnCl_2_ and FeCl_3_ as activator, displaying high specific capacitance of 361.6 F g^−1^ at a current density of 1 A g^−1^ [17]. 

In order to further improve the electrochemical performance of biomass carbon materials, heteroatom doping is another effective method. At present, a lot of heteroatom-rich biomass has been selected as precursors of porous carbon, such as flour [18], wheat bran [19], durian shell [20] and bean shell [21]. As a cost-effective and sustainable biomass, laver grows in the sea, which is rich in heteroatoms (N, O, S, Cl) that can be prepared to heteroatom-self-doping porous carbon. Moreover, the thallus of laver consists of a layer of cells embedded in a thin layer of glia, so the appearance of laver itself is fluffy and thin, which is beneficial to the preparation of hierarchical porous carbon. Herein, multiheteroatom-codoped laver-derived hierarchical porous carbons (BLHPC-Zn/K) have been obtained by copyrolysis synthesis with the KCl/ZnCl_2_ as a combined activator and porogen and H_3_BO_3_ as both a boron source and porogen. In the process of activation, impregnation and freeze drying were used to make the biological cells of laver fully contact with the activator, so that the layer was deeply activated. The as-prepared carbon exhibited 3D interconnected porous morphology, including macropores, mesopores and micropores with large specific surface area (SSA, 1514.9 m^2^ g^−1^) [22]. In the added mix salts, KCl as a cubic crystal can provide a template to obtain rich mesopores/macropores and ZnCl_2_ mainly generates rich micropores of carbon materials [23]. The addition of boric acid not only increases the mesopores and macropores of carbon materials, but the doping of boron also improves the conductivity and surface defects of carbon materials, thereby boosting performance of supercapacitors and lithium-ion batteries [14,24]. This work provides a strategy for producing environmentally friendly, large-scale, low-cost and high-performance carbon materials for energy-storage and conversion devices.

## 2. Materials and Methods

### 2.1. Materials

Laver was purchased from Fujian Fuchang Food Company (Fujian, China). ZnCl_2_, KCl, H_3_BO_3_, HCl, Na_2_SO_4_ and KOH were purchased from Sinopharm Chemical Regent Co., Ltd. (Shanghai, China). N-methyl-2-pyrrolidone (NMP), LiPF_6_, Polyvinylidene fluoride (PVDF) and 1-butyl-3-methylimidazolium tetrafluoroborate/acetonitrile (BMIMBF4/AN) electrolyte were purchased from Beijing Saibo New Chemical Materials Co., Ltd. (Beijing, China).

### 2.2. Materials Preparation

Typically, ZnCl_2_ (2.5 g), KCl (2.5 g) and H_3_BO_3_ (1.0 g) were dissolved in 50 mL distilled water; laver (0.5 g) was then dispersed in above mixture solution. Thereafter, the as-prepared mixture was stirred for 1 h and soaked overnight. After freeze drying, the fluffy samples were transferred to a quartz boat and heated from room temperature to 800 °C for 2 h under Ar atmosphere with a heating rate of 3 °C min^−1^ in a tube furnace. After cooling to room temperature, the obtained black solid was grinded and centrifugally cleaned with 1 M HCl solution and distilled water, then dried at 70 °C for 12 h in a vacuum oven, which is denoted as BLHPC-Zn/K. As comparisons, the LHPC-Zn/K, LHPC-Zn and LHPC-K were fabricated using the same procedure without H_3_BO_3_, KCl and H_3_BO_3_, ZnCl_2_ and H_3_BO_3_, respectively.

### 2.3. Characterization

The morphology of as-prepared samples was characterized by scanning electron microscopy (SEM, Zeiss Merlin Compact) and transmission electron microscopy (TEM, FEI Tecnai G2 F20). X-ray diffraction with Cu Kα radiation (λ = 0.15406 nm) (XRD, X’ Pert PRO) was used to analyze the crystal structure of the as-prepared materials. The Raman characterization was obtained with Renishaw inVa Raman microscope with an excitation wavelength of 785 nm. X-ray photoelectron spectroscopy (XPS) was investigated with a Thermo Scientific K-Alpha with Al Kα radiation 12.0 kV, 6 mA. The specific surface areas (SSAs) and pore-size distribution (PSD) were carried out by a nitrogen adsorption−desorption measurement at 77 K on a BET analyzer (ASAP2460). The SSA and PSD of samples were obtained based the Brunauer–Emmett–Teller (BET) method and density functional theory (DFT) method.

### 2.4. Electrochemical Characterization

The supercapacitive properties of the as-prepared samples were studied in a three-electrode cell with the electrolyte of 6 M KOH aqueous solution. The platinum foil and Ag/AgCl electrode were used as the counter and reference electrodes, respectively. The working electrodes were prepared by mixing the 80 wt% active materials, 10 wt% acetylene black and 10 wt% poly(tetrafluoroethylene) binder into slurry and pressing it onto the nickel-foam current collector (1 cm × 1 cm). The mass loading of the electrode materials was ~2 mg cm^−2^. The symmetric supercapacitor was assembled using 1 M Na_2_SO_4_ as the aqueous electrolyte and 1 M 1-butyl-3-methylimidazolium tetrafluoroborate/acetonitrile (BMIMBF_4_/AN) as the organic electrolyte. The single electrode was measured in KOH to better compare the performance of single electrode with the literature, and supercapacitors were tested in neutral electrolytes for future practical applications. The glassy fibrous paper (Whatman GF/G) was used as the separator. Cyclic voltammetry (CV), Galvanostatic charge–discharge (GCD) and electrochemical impedance spectroscopy (EIS) were performed on an electrochemistry workstation (CH Instruments CHI660E).

The gravimetric specific capacitance (F g^−1^) of single electrode was calculated according to the following equation [25]:

(1) $$Cm=\frac{I\times △t}{m\times △V}$$

where ***I*** (mA) is the discharge current, ∆***t*** (s) is the discharge time, ∆***V*** (V) is the voltage window, and ***m*** (mg) is the mass loading of active materials.

The gravimetric specific capacitance (F g^−1^) of full SC was calculated based on the following equation:

(2) $$C\mathrm{cell}=\frac{I\times △t}{(m_{+}+m_{\_})\times △V\mathrm{total}}$$

where ***I*** (mA) is the discharge current, ∆***t*** (s) is the discharge time, ∆***V**_**total**_* (V) is the voltage window of full SC, and the total mass loading (mg) of active materials in both positive (***m**_**+**_*) and negative (***m**_**-**_*) electrodes.

The power density *P*_cell_ (W kg^−1^) and energy density *E*_cell_ (W h kg^−1^) of symmetric SC were calculated according to the following equation [26,27]:

(3) $$P\mathrm{cell}=\frac{E\mathrm{cell}}{△t}\times 3600$$

(4) $$E\mathrm{cell}=\frac{1}{2}C\mathrm{cell}\times △V^{2}\times \frac{1}{3.6}$$

where ∆***t*** (s) is the discharge time and ∆***V*** (V) is the discharge voltage range.

The lithium-storage performance of the as-prepared samples was tested with CR2032 coin cells with 1 M LiPF_6_ in dimethyl carbonate, ethylene carbonate and diethyl carbonate (1:1:1, in volume percent) as electrolyte. Mixed 80 wt% of active materials, 10 wt% of acetylene black, and 10 wt% of polyvinylidene fluoride (PVDF) in ***N***-methyl-2-pyrrolidone (NMP) to form slurry and coated the as-prepared slurry onto copper foil, then dried it in a vacuum oven at 120 °C for 12 h. The round electrodes with a diameter of 12 mm were acted as the working electrode, lithium metal foil was used as the counter and reference electrode. CV was carried out by CHI660E workstation, and GCD experiments were tested on a battery test instrument (Land CT2001).

## 3. Results

**Figure 1:**
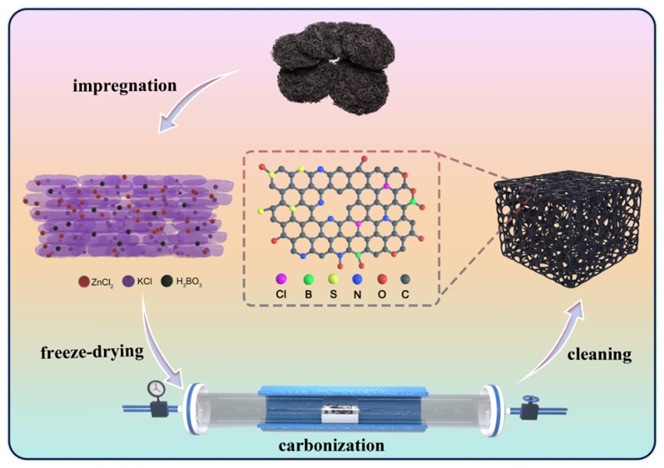
Schematic illustration for the fabrication process of biomass-derived carbon.

The fabrication process of biomass-derived carbon is shown in Figure 1. Herein, marine-product laver was chosen as precursor of carbon material, which is not only low-cost and environmentally friendly, but also rich in heteroatoms (N, S, Cl, O) to achieve heteroatom self-doping [28]. After the laver is soaked into activator solution, the activator ions are injected into the cells of biomaterials to prevent the easily occurring aggregating of the cell walls and to act as a supporter. Thus, the laver can be deeply activated in the carbonization process and obtain carbon materials with a rich pore structure. In this activation process, 800 °C was chosen as the optimal temperature for preparing carbon materials according to relevant literatures [13,18]. KCl/ZnCl_2_ mixed activator is applied to synthesize porous carbonaceous materials, which exhibits 3D interconnected porous morphology including macropores, mesopores and micropores with large specific surface area. Meanwhile, the addition of H_3_BO_3_ can provide boron doping as well as mesopores and micropores of carbon materials [29]. In addition, the activation method can obtain high carbon yield (about 20%), and the used activator can be recycled, and the porous carbon prepared by using the collected activator also has good electrochemical performance (Figure S1), which proves that this activation method is relatively practical.

**Figure 2:**
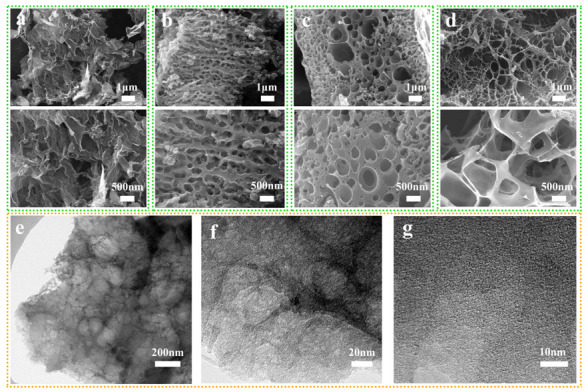
SEM images: (**a**) LHPC-K; (**b**) LHPC-Zn; (**c**) LHPC-Zn/K; (**d**) BLHPC-Zn/K; (**e**–**g**) the TEM images of the BLHPC-Zn/K.

The morphologies of LHPC-K, LHPC-Zn, LHPC-Zn/K, and BLHPC-Zn/K were revealed by SEM and TEM (Figure 2). Figure 2a shows porous activated carbon prepared with KCl as activator, which exhibits thin sheets of carbon with mesopores and macropores. After changing KCl into ZnCl_2_ activator, the porous morphology of the carbon material becomes obvious (Figure 2b). When mixed salt KCl /ZnCl_2_ is used as an activator, the porosity increases. Moreover, the activated carbon exhibits a hierarchical porous structure (Figure 2c). After introducing H_3_BO_3_, BLHPC-Zn/K presents unique 3D microstructure morphology (Figure 2d), which is beneficial to shorten the ions’ diffusion distance. The TEM (Figure 2e–g) confirms that the BLHPC-Zn/K sample has an interconnected hierarchical porous structure with mesopores, macropores and micropores; meanwhile, abundant micropores could be obviously observed in the high-resolution TEM image (Figure 2g). The interconnected hierarchical porous architecture provides highways for ion transport and further increases the storage capacity of carbon materials.

**Figure 3:**
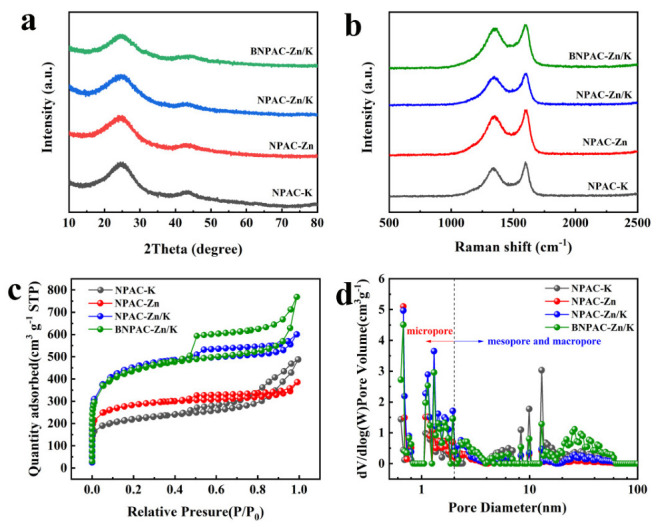
(**a**) XRD patterns; (**b**) Raman spectra; (**c**) the nitrogen adsorption–desorption isotherms and (**d**) pore-size distributions of MHPC-K, MHPC-Zn, MHPC-Zn/K, and BMHPC-Zn/K.

The XRD and Raman analysis was applied to explore the phase structures of as-prepared samples. As shown in Figure 3a, it presents two diffraction peaks, appearing at 25° and 43°, corresponding to the (002) and (101) lattice plane of graphite, which exhibits a typical feature of amorphous carbon [30]. The (002) diffraction peak is slightly deviated from graphite carbon (2θ = 26.5°, JCPDS No. 89-7213), suggesting that heteroatom doping leads to the expansion of the layer spacing. Moreover, the (002) peak of BLHPC-Zn/K is weakest and broadest among the four samples; it is suggested that abundant pore structure leads to a reduced graphitization degree. Figure 3b shows two peaks located at 1350 cm^−1^ and 1590 cm^−1^, referring to the D-band and G-band, respectively. The ID/IG intensity ratio is used to measure the degree of disorder in graphitic materials [31]. Among the four samples, the ID/IG value of BLHPC-Zn/K is highest, as shown in Table 1. This result suggests that the disorder of carbon structure increases after multiheteroatom doping, which is consistent with XRD.

The nitrogen adsorption–desorption isotherms and pore-size distributions reveal the pore structure of LHPC-K, LHPC-Zn, LHPC-Zn/K and BLHPC-Zn/K, presented in Figure 3c,d. The curves of all samples exhibit the combined type I and IV isotherms (Figure 3c). Obviously, a steep rise at a very low-pressure area (P/P_0_ < 0.1) suggests the existence of micropore and hysteresis loops at the pressure area (0.4 < P/P_0_ < 0.9) and a slight rise appearing in the high pressure (0.9 < P/P_0_ < 1) owing to characteristics of mesopores and macropores [32]. It is very clear that all samples have the hierarchical porous structure. Meanwhile, BLHPC-Zn/K exhibits the most apparent hysteresis loop, suggesting that it has a richer mesopore structure than the other three samples. The pore-size distribution is shown in Figure 3d. The pore size of BLHPC-Zn/K contains micropores (0.6–2 nm), mesopores (2–50 nm) and macropores (>50 nm), and BLHPC-Zn/K has the largest mesoporous peak intensity, implying that it has a higher content of mesopore structure. For the addition of a simplex salt as an activator, LHPC-K obtains richer mesopores and LHPC-Zn has abundant micropores. The specific surface area and pore volume of LHPC-K, LHPC-Zn, LHPC-Zn/K and BLHPC-Zn/K is summarized in Table 1; it is found that BLHPC-Zn/K has the largest specific surface area value of 1514.3 m^2^ g^−1^ and the highest total pore volume of 1.16 cm^3^ g^−1^ among the four samples. Beneficial from the mixed activator, the prepared activated carbon has a hierarchical porous structure and a high specific surface area. Moreover, the addition of boric acid makes the porous carbon produce more mesoporous and macroporous structures.

**Figure 4:**
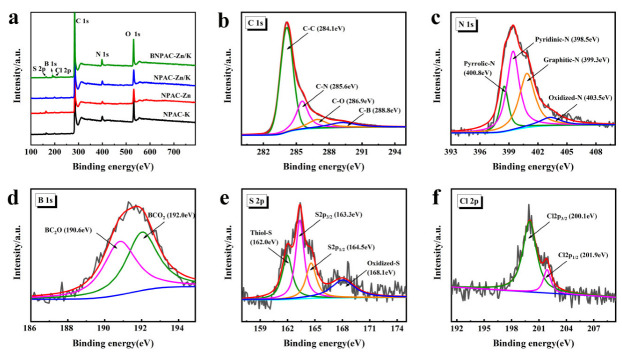
(**a**) The wide-scan XPS survey spectrum of LHPC-K, LHPC-Zn, LHPC-Zn/K and BLHPC-Zn/K; (**b**) C 1s spectrum; (**c**) N 1s spectrum; (**d**) B 1s spectrum; (**e**) S 2p spectrum; (**f**) Cl 2p spectrum of the BLHPC-Zn/K.

The surface chemical compositions of LHPC-K, LHPC-Zn, LHPC-Zn/K and BLHPC-Zn/K were analyzed via XPS. As shown in Figure 4a, all samples confirmed the presence of the characteristic peaks of C, N, O, S and Cl elements. Among them, the heteroatoms of N, O and S were from protein, fat and polysaccharide of laver. Moreover, as the laver is a typical marine product, the Cl elements also come from laver [28]. The characteristic peaks of B element can be observed in the XPS survey spectrum of BLHPC-Zn/K, suggesting the successful doping of B. The quantitative analysis of each element is summarized in Table 1, in which it can be found that BLHPC-Zn/K has higher nitrogen content than other samples. It is reported that B element can anchor N, which increases the content of N element [33,34]. Moreover, the O content of BLHPC-Zn/K is higher and the surface hydrophilicity has been enhanced, significantly promoting the infiltration of electrolyte to the electrode material. Based on the high resolution, the C 1 s peak can be divided into four peaks at 284.1, 285.6, 286.9 and 288.8 eV, corresponding to C-C, C-N, C-O and C-B, respectively (Figure 4b) [35]. N 1 s consists of four peaks located at 398.5, 399.3, 400.8 and 403.5 eV, correlating to pyridinic-N, graphitic-N, pyrrolic-N and oxidized-N, respectively (Figure 4c) [36]. Generally, pyridinic-N and pyrrolic-N can create defects and provide electroactive sites, which contribute to ion storage [37,38]. Moreover, graphitic-N is also beneficial for improving the electronic conductivity of carbon materials. B 1 s can be derived into two types at 190.6 eV and 192.0 eV, which are ascribed to BC_2_O and BCO_2_, respectively (Figure 4d) [39]. B doping can increase surface wettability and significantly shorten ion-transport paths. The S 2p spectrum presents four peaks at 162.0, 163.3, 164.5 and 168.1 eV, attributable to thiol-S, S 2p_3/2_ and S 2p_1/2_, and oxidized-S, respectively (Figure 4e) [40]. The high-resolution spectrum of Cl 2p can be fitted into Cl 2p_3/2_ (200.1 eV) and Cl 2p_1/2_ (201.9 eV), respectively (Figure 4f) [28]. Doping sulfur and chlorine atoms to sp^2^ carbon framework can enhance electronic conductivity and reduce charge-transfer resistance of the electrode materials. To sum up, multiheteroatom doping exhibits a synergistic effect, resulting in superior performances.

**Figure 5:**
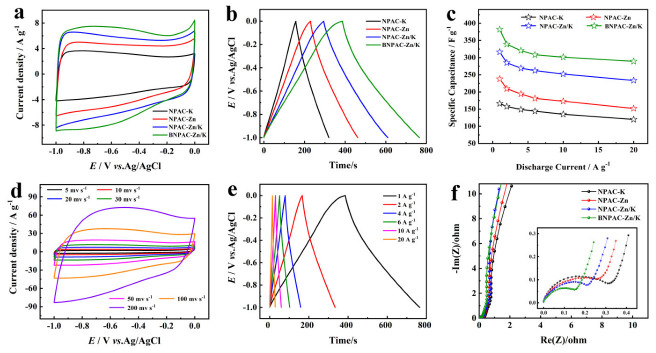
Supercapacitive performances of LHPC-K, LHPC-Zn, LHPC-Zn/K and BLHPC-Zn/K electrodes using 6 M KOH electrolyte in a three-electrode system: (**a**) CV curves at a scan rate of 20 mV s^−1^; (**b**) GCD curves at a current density of 1 A g^−1^; (**c**) The specific capacitances at various discharge-current densities; (**d**) CV curves and (**e**) GCD curves of the BLHPC-Zn/K electrode; (**f**) Nyquist plots.

The electrochemical performances of the LHPC-K, LHPC-Zn, LHPC-Zn/K and BLHPC-Zn/K electrodes for the supercapacitor were first measured in 6 M KOH electrolyte. Figure 5a shows the CV curves of all electrodes at a scan rate of 20 mV s^−1^, which exhibits a quasi-rectangle shape, indicating a typical capacitance characteristic of a double electric layer. Moreover, the CV curves of LHPC-Zn/K have more obvious humps than other electrodes; this is due to more heteroatom-doping content, which leads to the occurrence of pseudocapacitance. The BLHPC-Zn/K electrode exhibits the largest area of the CV curve, indicating the best specific capacitance. Figure 5b shows GCD curves at a current density of 1 A g^−1^, and the BLHPC-Zn/K electrode possesses the longest discharge time, which is consistent with the CV results. After calculating, the specific capacitances of the LHPC-K, LHPC-Zn, LHPC-Zn/K and BLHPC-Zn/K electrodes are 166.1, 237.7, 316.3 and 382.5 F g^−1^, respectively. Moreover, the BLHPC-Zn/K electrode exhibits superior rate capability (75.8%) with current densities from 1 A g^−1^ to 20 A g^−1^, which are higher than those of LHPC-K (72%;), LHPC-Zn (63.8%) and LHPC-Zn/K (73.6%) (Figure 5c). The ultrahigh specific capacitance and rate capability of BLHPC-Zn/K is attributed to the 3D interconnected porous structure with large specific surface area promoting rapid ion transport and heteroatom doping, especially B doping could boost the hydrophilicity of carbon and electronic conductivity. Furthermore, as shown in Table 2, the specific capacitance of BLHPC-Zn/K is higher than those of biomass carbon materials in recent references.

The CV curves of BLHPC-Zn/K at scan rates from 5 to 200 mV s^−1^ are displayed in Figure 5d. Obviously, the CV curves remain approximately rectangular-shaped, even at a high scan rate of 200 mV s^−1^, suggesting outstanding rate performance. GCD tests (1–20 A g^−1^) were conducted to further investigate the capacitive behaviors of BLHPC-Zn/K electrode. As shown in Figure 5e, the GCD curves retain nearly symmetrical features at all current densities, suggesting excellent reversibility. Figure 5f shows the Nyquist plots of the as-prepared electrodes, which all consist of a steep straight line in the low-frequency region, suggesting ideal capacitive performance, and a small semicircle in the high-frequency region, indicating low charge-transfer resistance (Rct). By contrast, BLHPC-Zn/K exhibits the lowest Rct (0.23 Ω) among the LHPC-K (0.37 Ω), LHPC-Zn (0.36 Ω) and LHPC-Zn/K (0.29 Ω), implying the fastest electrochemical reaction kinetic, which resulted from the synergistic effect of multiheteroatom doping, leading to high conductivity.

The relationship between the peak current (***i***) and the sweep rate (***v***) is based on the following equation:

(5) $$i=av^{b}$$

where ***a*** and ***b*** are adjustable parameters. The value of ***b*** is related to the charge-storage mechanism of the materials. The material is considered a diffusion-controlled process (***b*** = 0.5), or a capacitive-controlled process (***b*** = 1). The *b* values of the anodic peak and cathodic peak are 0.90 and 0.94 (Figure S2), suggesting the capacitive-controlled process of the BLHPC-Zn/K electrode. The major charge-storage mechanism of the BLHPC-Zn/K electrode is attributed to multiheteroatom doping (B, N, O, Cl and S), which can provide pseudocapacitance, thereby improving reversible capacity.

**Figure 6:**
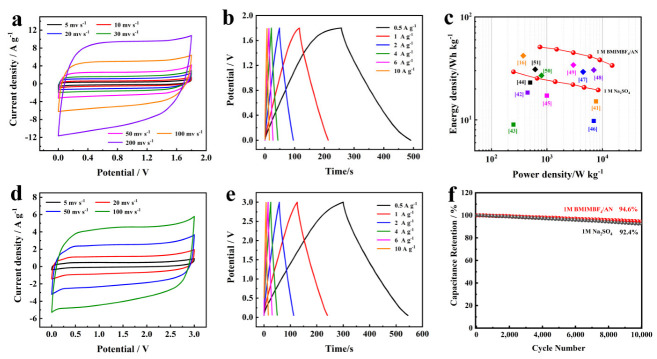
Electrochemical performance of BLHPC-Zn/K// BLHPC-Zn/K symmetric supercapacitor. CV curves in (**a**) 1 M Na_2_SO_4_ and (**d**) 1 M BMIMBF_4_/AN electrolytes; GCD profiles in (**b**) 1 M Na_2_SO_4_ and (**e**) 1 M BMIMBF_4_/AN electrolytes; Ragone plots in (**c**) 1 M Na_2_SO_4_ and1 M BMIMBF_4_/AN electrolytes; The cycling performance at a current density of 4 A g^−1^ in (**f**) 1 M Na_2_SO_4_ and 1 M BMIMBF_4_/AN electrolytes.

In order to further evaluate the supercapacitive performance of BLHPC-Zn/K electrode in practical application, the symmetric supercapacitors have been assembled in 1 M Na_2_SO_4_ as aqueous electrolytes and 1 M BMIMBF_4_/AN ionic liquid as organic electrolytes, the test results are shown in Figure 6. Obviously, the CV curves show a quasi-rectangular shape without phenomenon of hydrogen and oxygen evolution, indicating that the potential can be performed in the range of 0–1.8 V (Figure 6a and Figure S3). Figure 6b shows the GCD curves of the BLHPC-Zn/K//BLHPC-Zn/K symmetric supercapacitor at a current density from 0.5 to 10 A g^−1^, which exhibits highly linear symmetry with low IR drop (0.02 V at 0.5 A g^−1^), suggesting its outstanding reversibility and low internal resistance. Based on the GCD test results (Figure S4), energy density corresponding to power density was calculated, and Ragone plots are shown in Figure 6c. Notably, the symmetric supercapacitor in aqueous electrolytes possesses a maximum energy density of 29.2 W h kg^−1^ at a power density of 250 W kg^−1^, which is much larger than those reported previously (Table S1) [41,42,43,44,45,46]. The cycling performance at a current density of 4 A g^−1^ was exhibited in Figure 6f. After 10,000 cycle tests, the capacitance retention rate of the BLHPC-Zn/K//BLHPC-Zn/K supercapacitor could reach 92.4%, indicating excellent cycle stability.

**Table 2:** Comparison of the specific capacitance of BLHPC-Zn/K with other biomass carbon materials in a three-electrode system.

Materials	Activation Methods	Electrolytes	Working Window of Electrolyte	Mass Loading (mg cm^−2^)	Current Density (A g^−1^)	Capacitance (F g^−1^)	Ref.
laver	ZnCl_2_, H_3_BO_3_, KCl	6 M KOH	−1–0 V	2.0	1	382.5	this work
rapeseed cake	K_2_CO_3_, phytic acid, melamine	6 M KOH	0–1 V	-	0.05	358	[15]
peanut shells	CoCl_2_, ZnCl_2_	6 M KOH	−1–0 V	2.0	0.5	343	[16]
Walnut shell	H_3_PO_4_, KOH	6 M KOH	−1–0 V	1.2	0.5	332	[18]
bean shell	H_3_BO_3_	6 M KOH	−1–0 V	3.0	0.5	119	[21]
sedum spectabile stalk	CH_4_N_2_S, H_3_BO_3_, ZnCl_2_	6 M KOH	−1–0 V	3.0	0.5	290.7	[24]
glucose	ZnCl_2_, H_3_BO_3_, NaCl	3 M KOH	−1–0 V	2.7	1	379.9	[32]
fir bark	NH_4_B_5_O_8_4H_2_O	6 M KOH	−1–0 V	1.5	0.5	188	[40]
soybean straw	KOH	6 M KOH	−1–0 V	4.0	0.5	325	[41]
Rice straw	KHCO_3_	6 M KOH	−1–0 V	4.0	1	317	[45]

According to the calculation formula of energy density: *E* = *1/2CV^2^*, the energy density of supercapacitors can be improved by increasing the working voltage range and specific capacitance. The ionic liquid electrolyte (1 M BMIMBF_4_/AN) with wide operating voltage (0–3 V) has higher energy density than the aqueous electrolyte (1 M Na_2_SO_4_) with operating voltage of 0–1.8 V. For this reason, BMIMBF_4_/AN ionic liquid is further chosen as the electrolyte. Figure 6d shows the CV curves of BLHPC-Zn/K// BLHPC-Zn/K symmetric supercapacitor at scan rates from 5 to 100 mV s^−1^. Clearly, the voltage can be expanded to 3 V in the 1 M BMIMBF_4_/AN electrolyte, and the shape of CV curves is not deformed. The GCD test has been performed under the potential window of 0–3 V (Figure 6e), the result shows that the curve presents a high degree of symmetry, suggesting an ideal supercapacitor behavior (Figure S5). The Ragone plots of the BLHPC-Zn/K// BLHPC-Zn/K symmetric supercapacitor are presented in Figure 6c. Benefiting from the high potential window, the maximum energy density can reach to 51.3 W h kg^−1^ at a power density of 250 W kg^−1^; even at high power density (15 kW kg^−1^), the symmetric supercapacitor can provide an energy density of 34 W h kg^−1^. Such superior energy density and power density has an advantage over most of previously reported biomass-carbon-based symmetric supercapacitors (Table S2) [16,47,48,49,50,51]. Moreover, the symmetric supercapacitor delivers remarkable cycling stability with capacitance retention of 94.6% after 10,000 cycles at a current density of 4 A g^−1^, suggesting an excellent practical value (Figure 6f).

**Figure 7:**
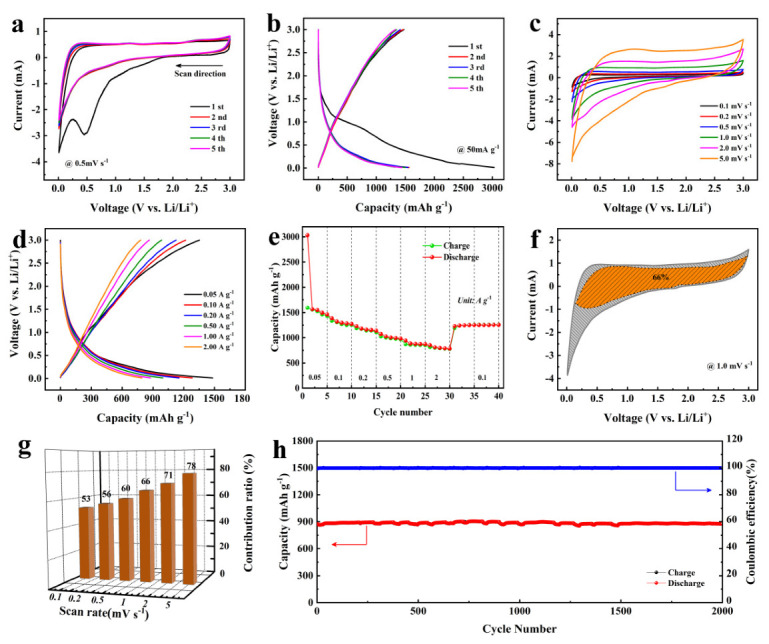
The storage performances of BLHPC-Zn/K. (**a**) CV curves at a scan rate of 0.5 mV s^−1^; (**b**) GCD curves a current density of 50 mA g^−1^; (**c**) CV curves at different scan rates; (**d**) GCD curves at different current densities; (**e**) The rate performance of the BLHPC-Zn/K; (**f**) CV curves of BLHPC-Zn/K with separation between total current and capacitive current (shaded region) at 1 mV s^−1^; (**g**) Capacitive contributions of the BLHPC-Zn/K at various scan rates; (**h**) cycling performance at 1 A g^−1^.

To investigate the Li-ion storage performances of BLHPC-Zn/K, the half-cell was assembled and measured within the voltage range from 0.01 to 3.0 V. Figure 7a exhibits the first five CV curves of BLHPC-Zn/K anode at a scan rate of 0.5 mV s^−1^. Obviously, a distinct peak (~0.5 V) appeared in the first negative curve, which is attributed to the formation of solid electrolyte film (SEI) and the consumption of Li ions [52,53]. Moreover, a wide peak has been found around 1.5 V, which corresponds to the reactions between Li ions and functional groups on the multi heteroatom doped carbon material surface. In subsequent cycles, the CV curves of BLHPC-Zn/K electrode gradually stabilized, suggesting an excellent cyclic stability. Figure 7b shows the GCD curves of BLHPC-Zn/K electrode at a current density of 50 mA g^−1^. Clearly, the specific capacity of the first cycle was larger than those other cycles, which was mainly due to the generation of SEI film and the consumption of electrolyte. The CV curves of the BLHPC-Zn/K electrode at scan rates from 0.1 mV s^−1^ to 5 mV s^−1^ are shown in Figure 7c. As the scan rate increases, the current response becomes larger, and the shape of the CV curve changes slightly, indicating a good reversibility. Moreover, the BLHPC-Zn/K electrode possesses high reversible capacity and excellent rate capability. As shown in Figure 7d,e, the BLHPC-Zn/K electrode achieved reversible capacities of 1497, 1282, 1154, 994, 876 and 791 mA h g^−1^ at current densities of 0.05, 0.1, 0.2, 0.5, 1 and 2 A g^−1^, respectively. Moreover, when the current density went back to 0.1 A g^−1^, the reversible capacity retention returned to 1256 mA h g^−1^, implying superior rate capability. In order to further study the lithium-storage mechanism of the BLHPC-Zn/K electrode, the method of Dunn et al. was used to analyze the diffusion-controlled and capacitive-controlled contribution ratios, according to the following formula [54,55]:

***i***(V) = ***k***_1_***v*** + ***k***_2_***v***^1/2^ (6)

where ***i***(V) is the current corresponding to a fixed potential, ***v*** is the scan rate, and ***k***_1_ and ***k***_2_ are constants, ***k***_1_***v*** an ***k***_2_***v***^1/2^ represent diffusion-controlled and capacitive-controlled contributions, respectively.

Based on this method, the capacitive contribution ratio of the BLHPC-Zn/K electrode was 66% at a scan rate of 1 mV s^−1^ (shaded region in Figure 7f). Moreover, the ratio of capacitive-controlled contribution increased from 53% to 78% at the scan rate from 0.1 to 5 mV s^−1^, indicating that the capacitive-behavior-dominant lithium storage of the BLHPC-Zn/K electrode (Figure 7g). The high ratios of capacitive contribution of the BLHPC-Zn/K electrode are attributed to the abundance of heteroatoms on the surface of carbon materials, especially nitrogen and boron, thus ensuring a high reversible capacity and an excellent rate capability. Cycling performance of the BLHPC-Zn/K electrode at 1 A g^−1^ is shown in Figure 7h. The reversible capacity of the BLHPC-Zn/K electrode saw no decay after 2000 cycles, and the coulombic efficiency reached to 100%, exhibiting a superior cycle performance.

The relationship between the above impressive electrochemical performance and the pore structure, specific surface area and element doping of carbon materials is summarized as follows: (1) The hierarchical porous structure, including macropores, mesopores and micropores of the BLHPC-Zn/K sample can promote the rapid transport of electrons and ions and make the electrolyte fully permeated, thereby improving charge storage; (2) The 3D interconnected skeleton structure as an electronic transmission highway can enhance electronic conductivity and obtain high rate capability; (3) The high specific surface area of the BLHPC-Zn/K sample can increase the number of active sites to store charges efficiently; (4) The abundant multiheteroatom (B, N, O, Cl and S) can not only provide pseudocapacitance, but also increase defects of carbon materials, which effectively enhance the electrochemical performance. (5) The synergetic effect between the 3D interconnected hierarchical porous structure and multiheteroatom doping is beneficial to achieve optimum performance in different electrolytes.

## 4. Conclusions

In summary, this work focuses on combining the advantages of a 3D interconnected hierarchical porous structure and heteroatom doping to improve the electrochemical performance of biomass-derived carbon materials. Here, the green synthetic methodes of impregnation, freeze-drying and carbonization are used to prepare porous carbon materials, in which KCl/ZnCl_2_ is a mixed activator and porogen, and the laver is chosen as a precursor to realize O, N, S, Cl self-doping. Moreover, the addition of boric acid introduces additional boron to the carbon material, which can increase the number of heteroatoms and achieve improved electrochemical performance of the carbon material. The obtained carbon material exhibits high surface area (1514.3 m^2^ g^−1^), 3D interconnected hierarchical porous structure and abundant heteroatom doping. As a result, the BLHPC-Zn/K electrode exhibits excellent specific capacitance of 382.5 F g^−1^ at 1 A g^−1^ and the assembled symmetric supercapacitor possesses a remarkable energy density of 51.3 W h kg^−1^ at a power density of 250 W kg^−1^ in 1 M BMIMBF_4_/AN. Moreover, the BLHPC-Zn/K sample as an anode for lithium-ion batteries shows a high reversible capacity of 1497 mA h g^−1^ at 1 A g^−1^ and superior cycling stability capacity (no decay after 2000 cycles). Thus, the BLHPC-Zn/K presents excellent performance in both supercapacitors and lithium-ion batteries, which will be a very promising material for practical application.

## Figures and Tables

**Table 1:** Surface areas, pore volume and compositions of LHPC-K, LHPC-Zn, LHPC-Zn/K and BLHPC-Zn/K, respectively.

Samples	*S*_BET_^a^ [m^2^ g^−1^]	*V*_total_^b^ [cm^3^ g^−1^]	*V*_micro_^c^ [cm^3^ g^−1^]	*ID/IG*	Elemental Analysis					
					C	O	N	S	Cl	B
LHPC-K	724	0.72	0.23	0.88	83.76	10.42	4.89	0.53	0.4	0
LHPC-Zn	925.7	0.6	0.36	0.89	85.8	9.01	4.19	0.65	0.35	0
LHPC-Zn/K	1394.6	0.92	0.49	0.9	85.27	9.58	4.32	0.4	0.43	0
BLHPC-Zn/K	1514.3	1.16	0.47	0.93	75.81	12.55	6.33	0.48	0.42	4.41

*S*_BET_^a^ is specific surface area based on BET equation. *V*_total_^b^ is the total pore volume determined from the nitrogen adsorption at pressure of 0.99. *V*_micro_^c^ is specific surface area of micropores obtained from t-plot method.

## Data Availability

Data can be made available upon request.

## Supplementary Materials

The following are available online at https://www.mdpi.com/article/10.3390/nano12101720/s1, Figure S1: (a) The schematic diagram of activator recovery process. The electrochemical performance of carbon materials prepared by using the collected activator: (b) CV curves at different scan rates, (c) GCD curves at different current densities, (d) the specific capacitance, Figure S2: The b value of specific peak currents with different scan rates, Figure S3: The CV curves BLHPC-Zn/K// BLHPC-Zn/K symmetric supercapacitor with different operation voltages recorded at the scan rate of 50 mVs-1, Figure S4: The specific capacitances of BLHPC-Zn/K// BLHPC-Zn/K symmetric supercapacitor in 1 M Na2SO4 electrolytes at various current densities, Table S1: Comparison of the energy density and power density of BLHPC-Zn/K// BLHPC-Zn/K with biomass carbon-based symmetric supercapacitors in aqueous electrolyte, Figure S5: The specific capacitances of BLHPC-Zn/K// BLHPC-Zn/K symmetric supercapacitor in 1 M BMIMBF4/AN electrolytes at various current densities, Table S2: Comparison of the energy density and power density of BLHPC-Zn/K// BLHPC-Zn/K with biomass carbon-based symmetric supercapacitors in ionic liquid electrolyte.
