# Supplementary material for: B, O and N Codoped Biomass-Derived Hierarchical Porous Carbon for High-Performance Electrochemical Energy Storage
Source: Nanomaterials (Basel). 2022 May 18;12(10):1720. doi: 10.3390/nano12101720 (PMC9143239; doi:10.3390/nano12101720)
Supplement: Supplementary file 1 [file nanomaterials-12-01720-s001.zip › nanomaterials-1709825-supplementary.pdf]

Article

B, O and N co-doped biomass-derived hierarchical porous carbon for high-performance electrochemical energy storage

Shuying Kong [1,*], Xinzhu Xiang [2], Binbin Jin [3], Xiaogang Guo [4], Huijun Wang [5],Guoqing Zhang [6], Huisheng Huang [7] and Kui Cheng [8,*]

[1] Chongqing Key Laboratory of Inorganic Special Functional Materials, College of Chemistry and Chemical Engineering, Yangtze Normal University, Chong qing404100, China; xiangxinzhu6666@163.com (X.X.), bbjin001@126.com (B.J.), guoxiaogang0528@126.com (X.G.), wanghj@yznu.edu.cn (H.W.), yzhanggq@163.com (G.Z.), h.s.huang@hotmail.com (H.H.)

[2] College of Engineering, Northeast Agricultural University, Harbin 150030, China

* Correspondence: kongshuying@126.com (S.K.), chengkui@neau.edu.cn (K.C.)

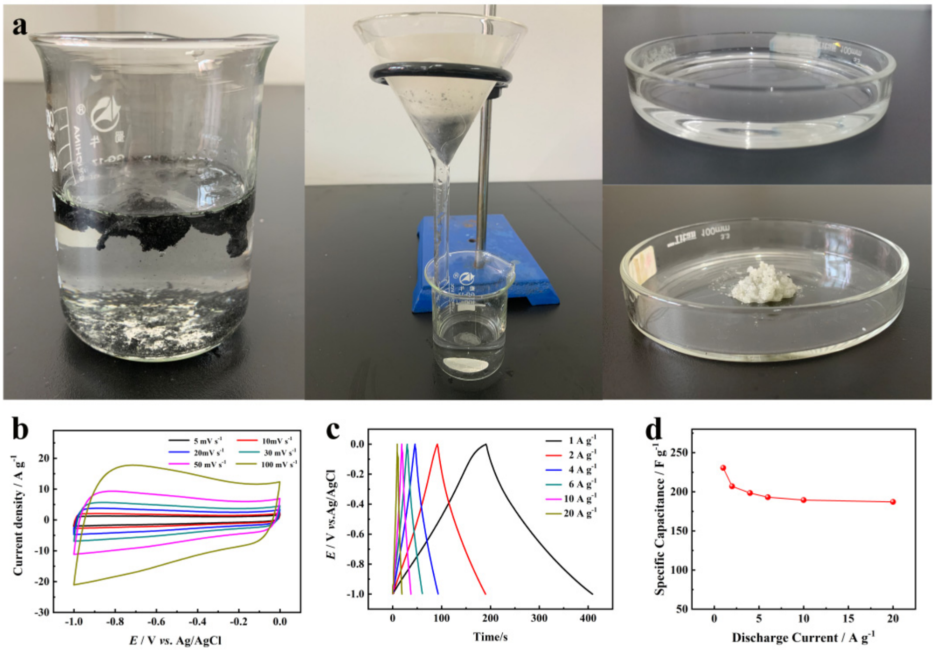

Figure S1. (**a**) The schematic diagram of activator recovery process. The electrochemical performance of carbon materials prepared by using the collected activator: (**b**) CV curves at different scan rates, (**c**) GCD curves at different current densities, (**d**) the specific capacitance.

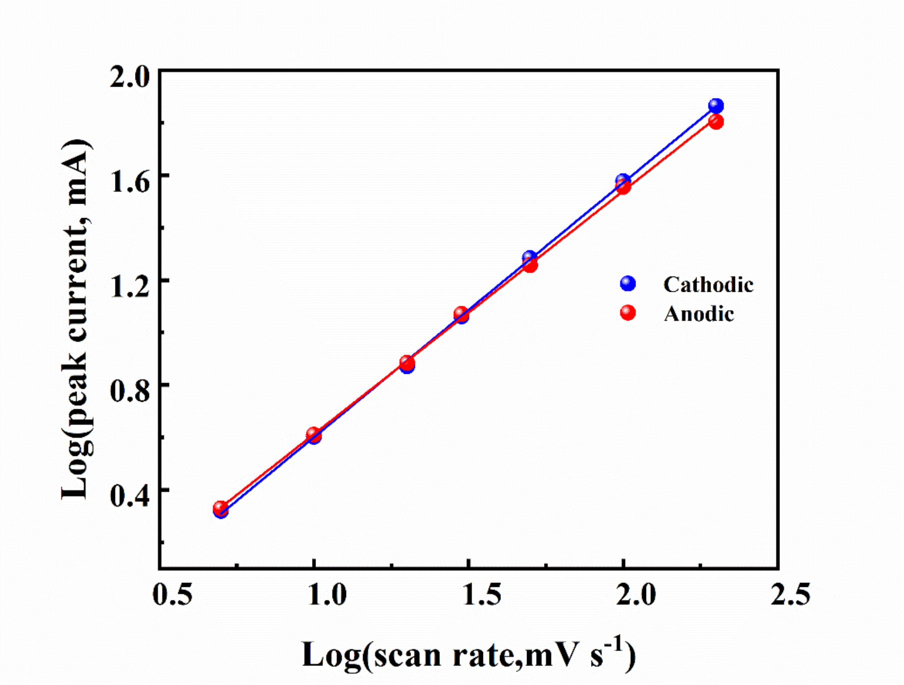

Figure S2. The *b* value of specific peak currents with different scan rates.

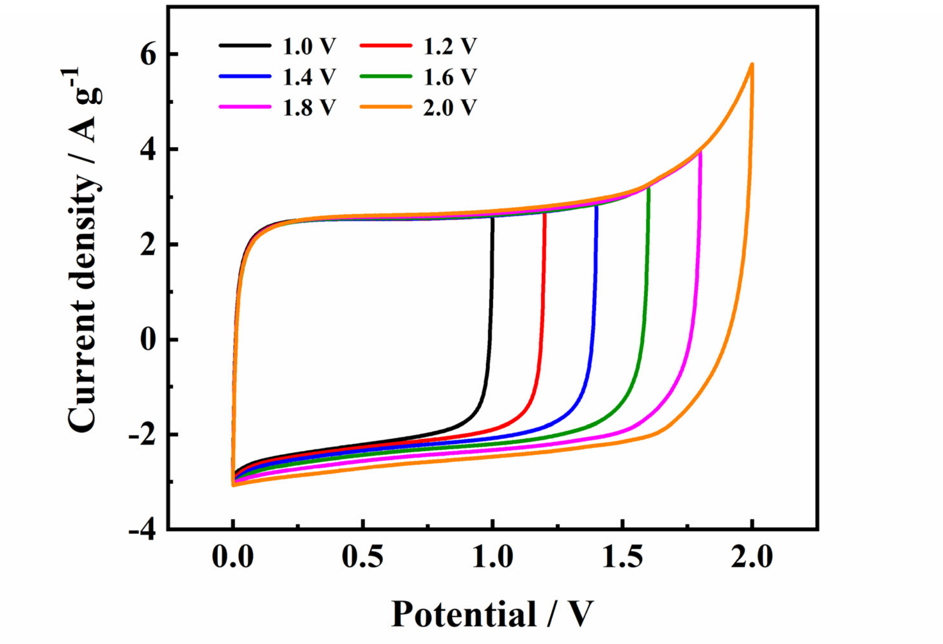

Figure S3. The CV curves BLHPC-Zn/K// BLHPC-Zn/K symmetric supercapacitor with different operation voltages recorded at the scan rate of 50 mVs$^{-1.}$

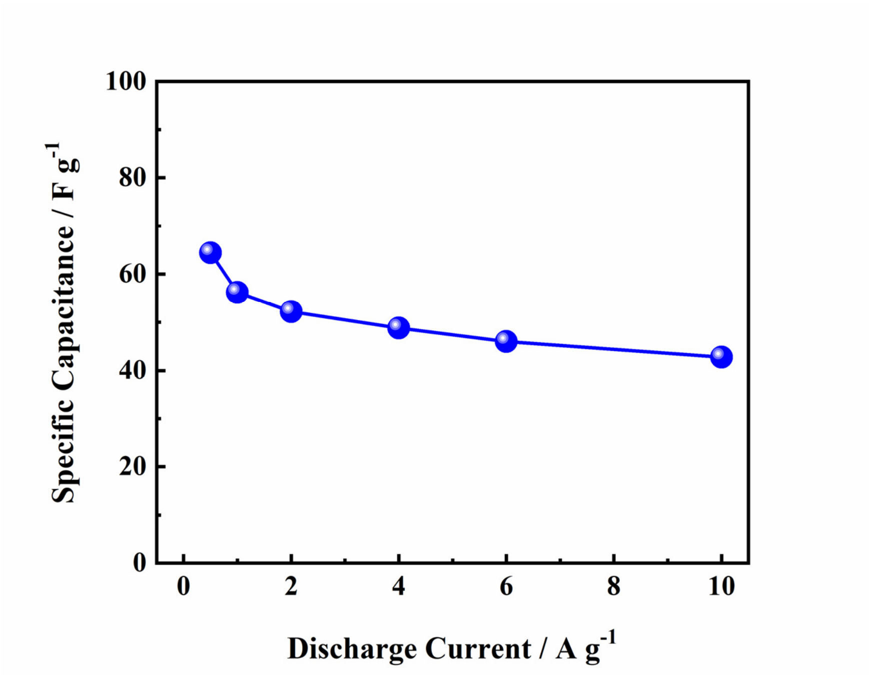

Figure S4. The specific capacitances of BLHPC-Zn/K// BLHPC-Zn/K symmetric supercapacitor in 1 M Na_2SO_4 electrolytes at various current densities.

Table S1. Comparison of the energy density and power density of BLHPC-Zn/K// BLHPC-Zn/K with biomass carbon-based symmetric supercapacitors in aqueous electrolyte.

Symmetric supercapacitor	Electrolyte	Voltage window	Power density (W h kg^{-1})	Energy density (W kg^{-1})	Ref.
BLHPC-Zn/K// BLHPC-Zn/K	1 M Na_2SO_4	0–1.8 V	250	29.2	this work
ZCH-PC// ZCH-PC	1 M Na_2SO_4	0–1.6 V	7700	15.1	41
AC-KB-M// AC-KB-M	1 M Na_2SO_4	0–1.6 V	450	18.4	42
PAN/Konjac-800-1// PAN/Konjac-800-1	PVA-KOH	0–1.6 V	250	9.0	43
SRC-850-4// SRC-850-4	1 M Na_2SO_4	0–1.6 V	500	23.0	44
PC-1-2// PC-1-2	6 M KOH	0–1.6 V	1000	17.2	45
WPC-3//WPC-3	6 M KOH	0–1.6 V	7000	9.7	46

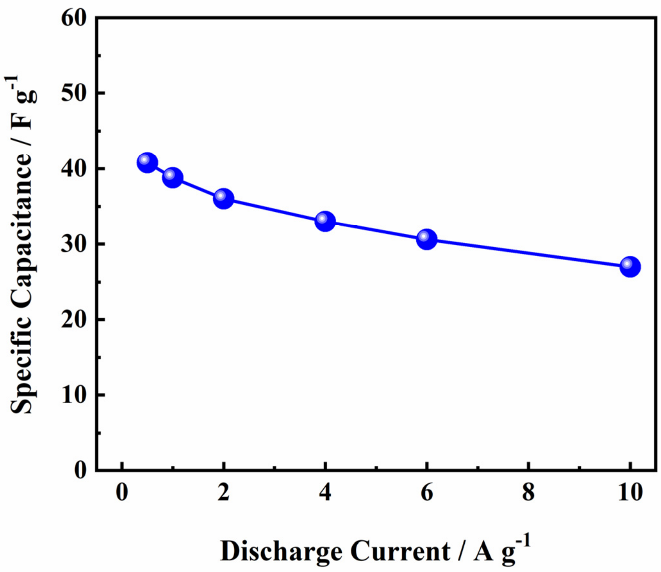

Figure S5. The specific capacitances of BLHPC-Zn/K// BLHPC-Zn/K symmetric supercapacitor in 1 M $BMIMBF_4$/AN electrolytes at various current densities.

Table S2. Comparison of the energy density and power density of BLHPC-Zn/K// BLHPC-Zn/K with biomass carbon-based symmetric supercapacitors in ionic liquid electrolyte.

Symmetric supercapacitor	Electrolyte	Voltage window	Power density (W h kg^{-1})	Energy density (W kg^{-1})	Ref.
BLHPC-Zn/K// BLHPC-Zn/K	1 M $BMIMBF_4$/AN	0–3.0 V	750	51	this work
NOHPPC-2// NOHPPC-2	1 M $BMIMBF_4$/AN	0–3.0 V	375	42	16
PAGC-2// PAGC-2	1 M $BMIMBF_4$/AN	0–3.0 V	4500	29.3	47
SLP-AC// SLP-AC	$EMIMBF_4$	0–3.0 V	7000	30.5	48
CCPF-750-15// CCPF-750-15	1 M $BMIMBF_4$/AN	0–3.0 V	3000	34	49
PBAC-600//PBAC-600	2 M [EMIM]BF_4/AN	0–2.6 V	800	27	50
GPCNF-SC//GPCNF-SC	1 M EMITFSI	0–3.5 V	616	30.9	51
